# Toxicity and Behavioral Effects of Amending Soils with Biochar on Red Imported Fire Ants, *Solenopsis invicta*

**DOI:** 10.3390/insects15010042

**Published:** 2024-01-08

**Authors:** Jiantao Fu, Mingda Qin, Yue Liang, Yinglin Lu, Yuxing An, Yanping Luo

**Affiliations:** 1 School of Plant Protection, Hainan University, Haikou 570228, China; fjtxmy@126.com (J.F.);; 2 Institute of Nanfan & Seed Industry, Guangdong Academy of Sciences, Guangzhou 510316, China; 3 College of Plant Protection, South China Agricultural University, Guangzhou 510316, China

**Keywords:** *S. invicta*, biochar, toxicity, behavior

## Abstract

**Simple Summary:**

*Solenopsis invicta* (Hymenoptera: Formicidae), also known as the red imported fire ant, is a social insect and a worldwide invasive pest. Biochar is a widely used soil amendment. In this work, we investigated the effects of varying soil biochar levels on irritability and contact toxicity as well as the behavioral and physiological changes in red fire ant behavior. High doses of biochar were toxic and repulsive to red fire ants, impairing their ability to walk, grasp, attack, and gather while also causing an increase in the activity of enzymes associated with oxidative stress. This study supports the prudent utilization of biochar as well as the prevention and management of red fire ants.

**Abstract:**

*Solenopsis invicta*, often known as the red imported fire ants (RIFAs), is a well-known global invasive ant species that can be found in agricultural, urban, and natural environments worldwide. Simultaneously, it also inhabits the soil. Biochar is generated by the pyrolysis of organic matter under high-temperature anoxic environments and widely used in agricultural ecosystems and soil amendment. However, to date, it remains unknown as to whether soil application of biochar has a negative effect on RIFAs. In our study, we investigated the toxicity and irritability effects of different amounts of biochar (0%, 1%, 2%, 5%, 10%, and 20%) introduced into the soil on red fire ants; upon comparison with the control soil (0% biochar), the application of 1%, 2%, and 5% biochar did not result in significantly different results. But the utilization of biochar at a concentration over 10% effectively repelled the RIFAs, resulting in their departure from the treated soils. High doses of biochar were able to cause death of red fire ants; the mortality rate of red fire ants reached 55.56% after 11 days of 20% biochar treatment. We also evaluated the effects of biochar on four behaviors of red fire ants, namely aggregation, walking, grasping, and attacking; 20% of the biochar treatment group reduced aggregation by 64.22% and this value was 55.22%, 68.44%, and 62.36% for walking, grasping, and attacking. Finally, we measured the activities of superoxide dismutase (SOD), peroxidase (POD), and catalase (CAT) enzyme activity and malondialdehyde (MDA) content in red fire ants; the results showed that the activities of the three enzymes increased with the increase in biochar addition, which indicated that a high dose of biochar induced oxidative stress in red fire ants. Our results indicate that biochar has the potential to cause toxicity and repel red imported fire ants (RIFAs) in a manner that is dependent on the concentration. We propose that biochar could be utilized in the control and manufacturing of baits for red fire ant management. This work establishes a foundation for the prevention and management of red fire ants and the logical utilization of biochar.

## 1. Introduction

The red fire ant (*Solenopsis invicta* Buren), native to South America, is an invasive alien species originating from South America. It has been listed as one of the top 100 most harmful invasive species worldwide by the International Union for Conservation of Nature [1]. This ant species poses a serious threat to agroforestry production, public safety, ecosystems, and human health in regions where it has invaded [2]. The range of invasion by RIFAs in China is extending as a result of factors such as environmental changes and human activities. Consequently, the extent of damage is increasing significantly. Human activities helped facilitate the transportation of this species to 435 counties and districts in 12 provinces. This has primarily been achieved through the movement of commodities on ships, trains, and trucks [3]. As it invades new areas, it manifests in direct and indirect affecting native crops; it not only feeds on agricultural commodities but it also feeds on fruits, seeds, and plant roots, resulting in significant yield losses [4]. In addition, it has the ability to construct nests in both outdoor and indoor electrical apparatus, such as street lights, telephone switchboards, network cable converters, power supplies for irrigation equipment, water pumps, air conditioners, airport lights, traffic signals, and other public facilities and equipment, chewing up wires, cables, dams, agricultural machinery, and various public facilities and equipment. This species has a tendency to gnaw on wires, cables, dams, agricultural machinery, and other public facilities and equipment, which can lead to electrical wire short-circuits or equipment malfunctions. Consequently, this poses safety risks and potential threats [5]. Red imported fire ants’ rapidly increasing populations have an extensive effect on other arthropods, particularly beneficial insects that they prey on at all life stages, including eggs, larvae, pupae, and adults [2]. Furthermore, the aggressive behavior of *S. invicta* poses a danger to humans residing within the vicinity of their foraging sites, including parks and farmlands. The *S. invicta* stings typically induce pruritus and, within a few hours of the sting, a white papule may manifest. Individuals with heightened sensitivity may experience symptoms such as an elevated body temperature, skin rash, severe physiological response, or potentially fatal outcome [5]. Despite numerous attempts to avoid and manage red fire ants, their impact remains constrained and their invasive range continues to increase.

Biochar is a black carbon-rich porous solid material; it is generated by the process of organic waste pyrolysis, which involves heating organic materials in the absence of oxygen under controlled thermal conditions in the presence of limited oxygen under specific thermal combustion conditions [6]. A variety of plant components, animal waste, or even industrial waste can be used to produce biochar. Biochar is a versatile substance with numerous applications due to its unique chemical, physical, and biological properties. It is frequently employed in soil remediation, soil improvement, soil carbon sequestration, and fertilizer carrier [7]. It is crucial to be aware of the extent to which biochar-amended soil might harm organisms, as the inadvertent usage of a substance containing hazardous compounds can lead to ecological imbalance and, ultimately, ecosystem impoverishment. Prior studies have demonstrated that biochar has diverse impacts on microorganisms, vegetation, and soil invertebrates. A study found that biochar-amended soil had a higher bacterial diversity compared to soil without biochar [8]. Biochar extracts have also been found in several studies to have an inhibitory effect on bacteria [9]. There have also been several studies on the toxicity of biochar to earthworms, most of which have been conducted in contaminated soils where biochar has been used to reduce the toxicity of the soil, or in natural soils. Some studies have indicated, however, that excessive application of biochar to soil can have a detrimental effect on earthworms, potentially causing toxicity. Elliston and Oliver performed an earthworm avoidance experiment on *Eisenia fetida* using both natural soil and artificial soil [10], following a duration of more than two weeks. *Eisenia fetida* individuals were exposed to soil containing a 20% biochar amendment; as a result, alterations were observed in the structures of certain organisms, indicating a harmful impact of elevated biochar application rates on the test subjects. Although the advantages of incorporating biochar into the soil are apparent, its impact on soil organisms seems to be varied. So, it is imperative to assess the inclination of soil arthropods with high mobility towards soil treated with biochar.

Soil serves as the fundamental substrate for the sustenance and growth of terrestrial life. Microorganisms and soil animals are biological components that have an impact on the nutritional content of soil. Ants are the primary species of arthropods found in soil and they are referred to as “soil ecosystem engineers” [11]. They possess the capacity to modify the physical and chemical properties of soil and regulate its fertility. The red fire ant is a kind of social insect and a serious destructive invasive organism in the world. Studies have shown that red fire ant nesting has different effects on soil physicochemical properties [12]. In our study, we chose *S. invicta* as the test organism. We focused on its selection and tolerance by establishing varying amounts of biochar in the soil; the behavioral regulation effect and enzyme activity were also tested. It should be emphasized that this is probably the first literature on the effects of biochar on RIFAs. This study is expected to provide support for the control of red fire ants and the application of biochar.

## 2. Materials and Methods

### 2.1. S. invicta for Testing

The red fire ants used in the trial were collected from the east district of the Institute of Nanfan and Seed Industry, Guangdong Academy of Sciences, Guangzhou, Guangdong Province, China. Plastic buckets with a capacity of 18 to 20 L were used as the designated receptacle for gathering red fire ant colonies in the field. Plastic buckets with a capacity of 18 to 20 L were used as the designated receptacle for gathering red fire ant colonies in the field. Additionally, for the safety of the collectors, the handles of shovels, rubber gloves, and water shoes were coated with talcum powder to deter the red fire ants from climbing upwards. To locate red fire ant nests, shovels were employed to excavate the mounds situated on the ground or the subterranean portions of the nests. They were then rapidly placed into the prearranged plastic containers. The weeds or branches were promptly cut to prevent the ants from ascending to the rim of the bucket. Talcum powder was applied to both the interior and exterior of the bucket after collection. After retrieving the nests from the field, the colonies were separated and obtained using the drip method [13]. The separated colonies were kept at a temperature of 26 ± 2 °C, relative humidity (60 ± 10)%, and photoperiod of 14:10 (L:D) and were fed with *Tenebrio molitor* Linnaeus (Coleoptera: Tenebrionidae) and 10% sugar water. After 7 d of in-house rearing, healthy and active worker ants of uniform size with an average head width of 0.8 ± 0.1 mm and average body length of 3.0~4.0 mm were used for the test. All red fire ants for the experiment originated from the same colony.

### 2.2. Biochar and Soil Preparation

Sugarcane bagasse was subjected to pyrolysis at temperatures ranging from 500 to 600 degrees Celsius in the absence of oxygen in order to generate biochar, which was subsequently purchased from Henan Zhongxin Lantian Environmental Protection Equipment Co. in Nanyang, China.

In the experiment, the soils were obtained from the experimental field of the Institute of Nanfan and Seed Industry, Guangdong Academy of Sciences, Guangzhou, China. After being air-dried, the soils were filtered through a sieve with a 20-mesh opening before being combined with biochar. Both the soil and the biochar were weighed separately prior to the treatment and then five different biochar application treatments were carried out. These treatments consisted of applying 0% (control), 1%, 2%, 5%, 10%, and 20% biochar to the soils in proportion to their dry weight. In total, 100 g of sample for each treatment was used and this was repeated 3 times. After the soil had been treated with biochar, we added some distilled water at a rate of 100 mL/kg. The soil that had been treated was then employed in the bioassay after being incubated inside for a period of twenty days. Once every 5 days throughout the incubation period, an amount of distilled water equal to 50 mL/kg was supplied.

### 2.3. Scanning Electron Microscope (SEM) Observation of Biochar

The micropore structure of biochar was tested by SEM (TESCAN MIRA 3 XMU). The dried biochar particles were evenly spread on the conductive adhesive of the sample holders and the unadhered charcoal particles were blown off the conductive adhesive using a wash ball. The prepared sample holders were sprayed with gold in a vacuum environment and plated with a conductive film layer; then, the sample holders were placed in a scanning electron microscope for observation.

### 2.4. Irritability Effect Bioassay

Approximately 20 g of biochar-amended soil was placed in the bottom of a disposable lunch box (volume = 1000 cm^3^) and 20 g of control soil was placed on the opposite side, with the vertical walls of the lunch box coated with fluon emulsion to prevent the escape of red fire ants. In the experiment, 0%, 1%, 2%, 5%, 10%, and 20% biochar amended soil groups were set up and 0% treatment groups were used as controls. All of these treatments were replicated three times; 30 worker ants were deployed per treatment. We found that ants preferred to live in the soil without the biochar compared with the soil amended with biochar. A 2 mL centrifuge tube containing 10% sucrose solution was placed for water, while ham hocks were provided as the food source. The numbers of ants on both sides and the same replicate were counted at 1, 2, 3, 5, 7, 9, and 11 day after treatment and the irritability effect was subsequently calculated. The following equation was used to determine the repellency effect:

Irritability effect (%) = (N_c_ − N_t_)/N_c_ × 100 (1)

where N_c_ is the number of ants in control group and N_t_ is the number of ants in the treatment group.

### 2.5. Insecticidal Activity Bioassay

Approximately 40 g of biochar-amended soil (0%, 1%, 2%, 5%, 10% and 20%) was placed in a disposable lunch box with a vertical wall that was coated with fluon emulsion. The mixture was let to dry for 24 h to prevent the ants from getting out. A total of 30 workers were weighed and then positioned at the bottom of the beaker, all treatments were replicated there time. Each treatment was placed in a 2 mL centrifuge tube containing a 10% sucrose solution for water, while ham hocks were provided as the food source. The number of ants dead in each treatment group was counted at 1, 2, 3, 5, 7, 9, and 11 day after treatment. Cumulative mortality was also calculated at the same time.

For the final step, on the 11th day, the weights of each group of 30 red fire ants were weighed together and recorded for assessing changes in body weight. The workers were maintained at 25 ± 1 °C and a relative humidity of 80%. We used the following equations:

Accumulated mortality (%) = N_d_/N_t_ × 100 (2)

where N_d_ is the number of dead ants and N_t_ is the number of treatment ants.

### 2.6. Effects of Biochar-Amended Soil on the Behavior of S. invicta

**Figure 1:**
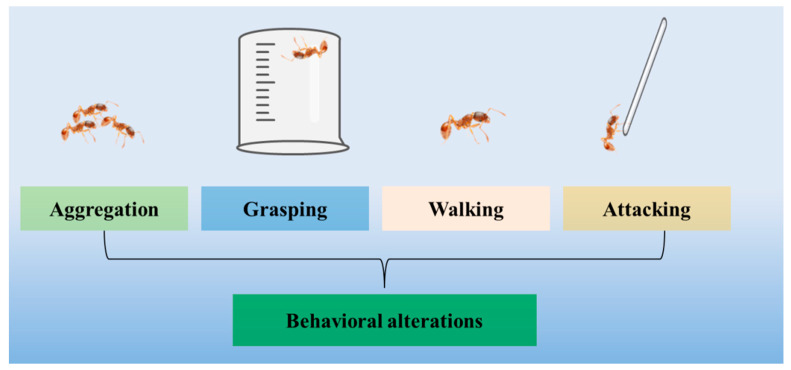
Behavioral observation test schemas for RIFAs.

Ants were handled in the same manner as those mentioned above in the insecticidal activity assay section. Four kind behaviors related to the survival and social activities of red fire ants, namely aggregation, aggression, grasping, and walking, were used for testing after 1, 2, 3, 5, 7, 9, and 11 day treatment, 30 worker ants were deployed per treatment and replicated three times. The behavioral observation test schemes are shown in Figure 1 and the detailed methodology is described as follows:

#### 2.6.1. Aggregation

The aggregation rate was evaluated in light of the previous report, with minor changes [14]. The number of aggregated red fire ants observed was recorded at 1, 2, 3, 5, 7, 9, and 11 day after treatment. Aggregation is considered possible for ants that can form a group of more than three (including three). Calculate the aggregation rate according to the formula:

Aggregation rate (%) = N_A_/N_t_ × 100 (3)

where N_A_ is the number of ants that can aggregate and N_t_ is the number of treatment ants.

#### 2.6.2. Attack

The attack rate was determined by making small adjustments to the prior report [15]. A slender wire was employed to consistently provoke the RIFAs; ants that bite the wire and fail to release it when lifted gently are classified as aggressive. Deceased ants are regarded as non-aggressive. The population of aggressive ants was monitored and documented at 1, 2, 3, 5, 7, 9, and 11 day following the treatment. The attack rate was determined by utilizing the following prescribed formula:

The attack rate (%) = N_A_/N_t_ × 100 (4)

where N_A_ is the number of ants that can attack and N_t_ is the number of treatment ants.

#### 2.6.3. Gripping

The assessment of the gripping rate was conducted based on a prior study [16]. The plastic cup was gently shaken after the red fire ants were placed at the bottom and left undisturbed for a minimum of 10 s. A sheet of blank paper was tightly positioned over the opening of the cup and then the cup was rotated 180°, positioning the mouth in a downward direction in order to cause the ants to descend onto the paper. Subsequently, we further halted for a minimum of 5 s; the cup expeditiously was then inverted and restored to its initial orientation. The number of RIFAs that dropped into the cup was monitored and documented. Ants that were unable to be gripped are the ones that ultimately ended up falling into the cup. Red fire ants that died are regarded as having been incapable of grasping. The number of ants engaged in grasping behavior was observed and documented at intervals of 1, 2, 3, 5, 7, 9, and 11 day following the treatment. The grasping rate was determined using the prescribed formula:

Grasping rate (%) = (N_t_ − N_f_)/N_t_ × 100 (5)

where N_t_ is the number of ants that can attack and N_f_ is the number of ants falling into the cup.

#### 2.6.4. Walking

Referring to the published literature [17], the red fire ants were placed on the white paper. If the red fire ants could walk for 5 s without falling down, they were considered to have the ability to walk. Calculation formula for the walking rate:

Walking rate (%) = N_w_/N_t_ × 100 (6)

where N_w_ is the number of ants that can walk and N_t_ is the number of red fire ants in all treatments.

### 2.7. Enzyme Activity Test

After a 7-day treatment with 5% biochar-amended soil, 0.1 g of red fire ants were weighed and added to 1 mL of the extraction solution, homogenized in an ice bath, centrifuged at 4 °C × 12,000 r/min for 10 min, and the supernatant was removed and utilized for conducting tests to determine the activity levels of superoxide dismutase (SOD), peroxidase (POD), malondialdehyde (MDA), and catalase (CAT). The biochemical enzyme activities were assessed using commercial assay kits (G0101F, G0107F, G0109F, and G0105F) obtained from Grace Biotechnology Co., Ltd., located in Suzhou, China.

The measurement of SOD activity involved the reduction of nitrogen blue tetrazole to produce blue formazan, which exhibits maximum absorbance at 560 nm.

The measurement of POD activity involved monitoring the alteration in absorbance at 470 nm, relative to a blank, caused by the production of 4-o-methoxyphenol resulting from the interaction between H_2_O_2_ and o-methoxyphenol.

The measurement of CAT activity was determined by assessing the rate at which H_2_O_2_ decomposes, which is directly related to its reduction at its distinctive absorption peak at 240 nm.

MDA, a byproduct of the oxidation of lipids by peroxidation, is commonly regarded as an indicator of lipid peroxidation. The method for assessing MDA levels involves measuring the response of MDA and other compounds that react with thiobarbituric acid, known as thiobarbituric acid reactive substances (TBARS). The concentration of MDA was measured by assessing the absorbance at 532 and 600 nm of the product trimethine (3,5,5-trimethyloxazole-2,4-dione) following the reaction of MDA with thiobarbituric acid (TBA), with adjustments made to account for the presence of sucrose as an interfering material.

### 2.8. Statistical Analyses

The statistical divergences were examined utilizing SPSS 22.0 software (Armonk, NY, USA). The independent samples *t* test was employed to examine the disparities between the two treatment groups, Duncan’s test was utilized to investigate the differences across six treatment groups. A significance level of *p* < 0.05 was regarded as significant [18]. The items labeled with the same letter indicate that there are no significant differences. The software known as origin 2021 was utilized in order to plot the graphs.

## 3. Results

### 3.1. SEM of Biochar

**Figure 2:**
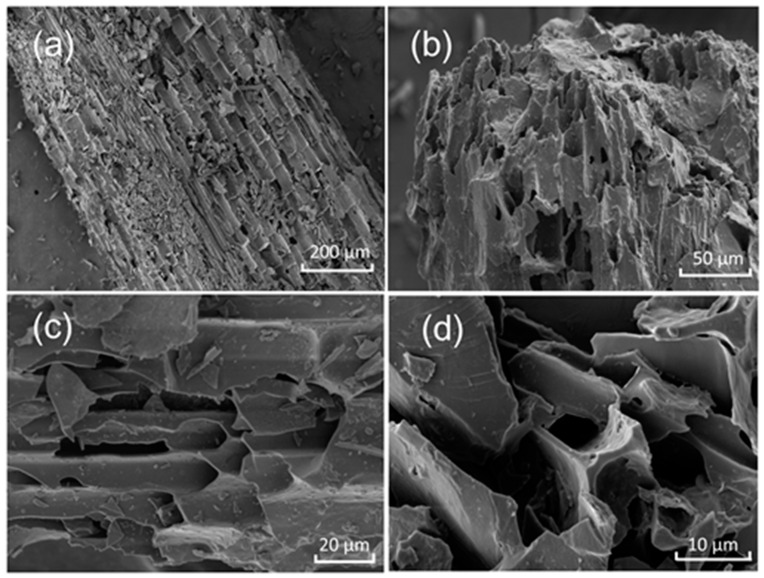
SEM images of the bagasse-derived biochar. (**a**) SEM with X200; (**b**) SEM with X1000; (**c**) SEM with X2000; (**d**) SEM with X5000.

The SEM images of biochar were presented in Figure 2, the biochar basically maintains the original shape of bagasse and the surface of both biochar was rough with a repeating pore structure (Figure 2a,b). The surface of bagasse biochar is relatively smooth and shows a regular porous block structure (Figure 2c,d).

### 3.2. Effects of Soil Amendments with Biochar on the Irritability Effect of RIFA

**Figure 3:**
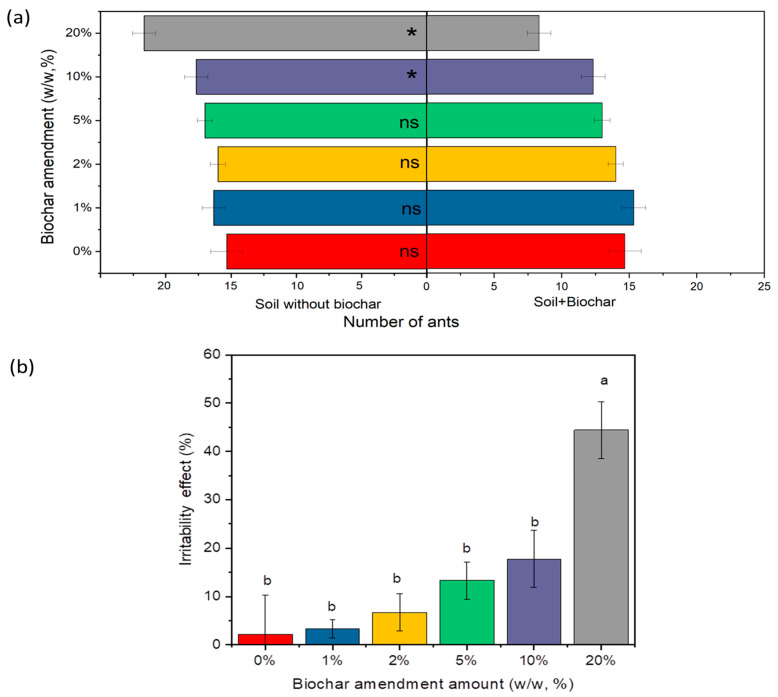
Irritability effect of *S. invicta* after 11 days of exposure to different amounts of biochar. (**a**) Ants numbers in each treatment in the irritability experiment. The * indicates a significant difference and ns indicates no significant difference between left and right compared treatment at *p* = 5%. (**b**) Irritability rate of 20% biochar treatment for 11 days. The ‘0’ on the x-axis was the control, which is soil without biochar. Data are presented as the mean ± standard error (S.E.). Different letters above bars indicate significant differences in the irritability effect among treatments due to concentration effects at the *p* < 0.05 level based on Duncan’s honestly significant difference (HSD) test (*n* = 3).

The results of the irritability test on red fire ants by biochar are shown in Figure 3. At 11 d after treatment, the number of red fire ants in 10% and 20% biochar-treated soils was significantly lower than that in the control soil and the differences between the 1%, 2% and 5% biochar treatments and the control group were not significant (Figure 3a). We also evaluated the repellency efficiency after 11 days of treatment using equation 1; the results showed that the repellency efficiency gradually increased with the increase in the amount of biochar, that the differences in repellency efficiency were not significant in the 1%, 2%, 5%, and 10% treatment groups, and that they were significant in the 20% treatment group compared with the control group (Figure 3b).

### 3.3. Effects of Soil Amendments with Biochar on the Insecticidal Activity of RIFA

**Figure 4:**
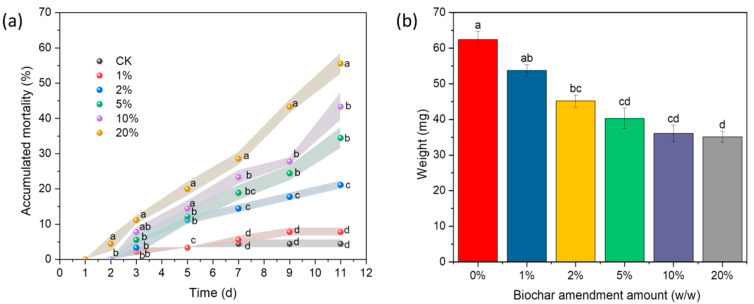
Toxic effects of soil amendments with different amounts of biochar on the RIFAs. (**a**) Cumulative mortality rate. The different letters among different treatments indicate significant differences at the same time, which were analyzed by Duncan’s test (*n* = 3 and *p* = 0.05). (**b**) Ants’ weight of soil treated with different amounts of biochar for 11 days. The ‘0’ on the x-axis was the control, which is soil without biochar. Different letters above bars indicate significant differences. Data are presented as the mean ± standard error (S.E.).

The soil amended with biochar was tested by RIFAs in order to investigate the effects of biochar on the lethal activity. The findings are illustrated in Figure 4. Observably, the cumulative mortality exhibited notable variations depending on the respective amount of biochar used (ranging from 0% to 20%) during an 11-day period of contact treatment (Figure 4a). A mortality rate of 55.56% was evident when the RIFAs after 11 days of soil addition of 20% biochar exposure treatment. The mortality rates gradually declined with the biochar amount decrease and became only 7.78% at the scale of 1% after 11 days. In the same number of biochar treatment groups, the mortality rate increased with the increase in time. However, there was no significant difference between the 1% treatment group and the control group. In addition, after 11 d, the groups in each treatment lost weight compared to the control group, with 20% of the treated groups losing 30.39% of their body weight after 11 d (Figure 4b).

### 3.4. Effects of Soil Amendments with Biochar on the Behavior of RIFAs

**Figure 5:**
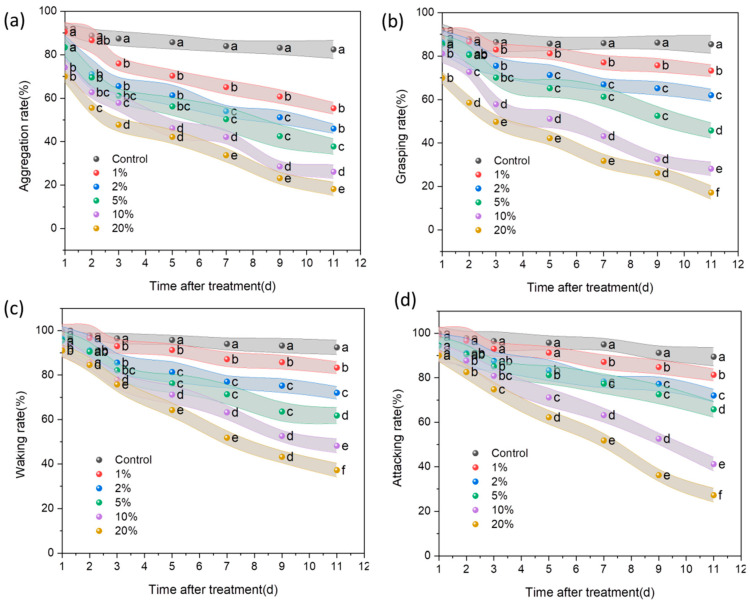
Effects of soil amendments with biochar on the behavior of RIFAs. (**a**) Accumulation rate. (**b**) Gripping rate. (**c**) Walking rate. (**d**) Attacking rate. The ‘0’ on the x-axis was the control, which is soil without biochar. Data are presented as the mean ± standard error (S.E.). The different letters among different treatments indicate significant differences at the same time and same concentrations, which were analyzed by Duncan’s test (*n* = 3 and *p* = 0.05).

Figure 5 depicts the results of an experiment in which the behavior alterations affect soil amendments with biochar on ants’ aggregation, walk, grasp, and attack rates for 11 days. After 11 days, the red fire ant aggregation rate was 82.44% in the control group, while it was only 18.22% in the 20% biochar-treated group (Figure 5a). Similarly, the red fire ant grasping rate of 20% biochar treatment for 11 d was 17.18 while it was 85.44% in the control group (Figure 5b). Similar results were found for the walking rate and attack rate (Figure 5c,d), where the behavior of red fire ants in the treated group was severely restricted. In addition, Biochar affected ant behavior concentration-dependently and time-dependently.

### 3.5. Effects of Biochar on Activities SOD, POD, CAT, and MDA in RIFAs

**Figure 6:**
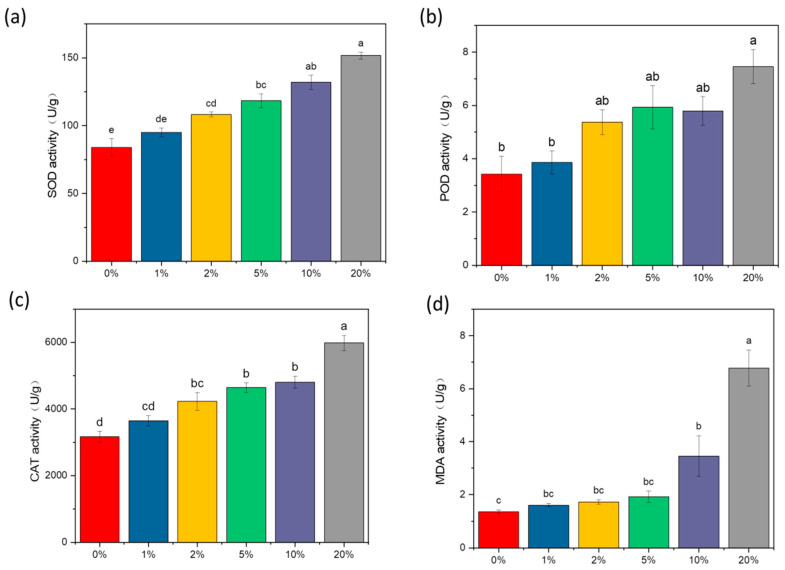
Changes in enzyme activity related to oxidative stress and MDA induced in the red fire ant after 5 d of 0%, 1%, 2%, 5%, 10%, and 20% biochar soil treatment. (**a**) SOD. (**b**) POD. (**c**) CAT. (**d**) MDA. The ‘0’ on the x-axis was the control, which is soil without biochar. Data are presented as the mean ± standard error (S.E.). The different letters among different treatments indicate significant differences, which were analyzed by Duncan’s test (*n* = 3, *p* = 0.05).

This experiment examined the effects of soil amendment biochar on the activity of SOD, CAT, and POD enzymes and MDA content in RIFAs (Figure 6). The results showed that the SOD enzyme content gradually increased with the increase in biochar treatment, with non-significant differences between the 1% treatment group and the control and significant differences between the 2%, 5%, 10%, and 20% treatment groups and the control (Figure 6a). Similarly, we observed that red fire ant POD enzyme activity increased with the amount of biochar but the differences were not significant between the 0%, 1%, 2%, 5%, and 10% treatment groups and significant between the 20% treatment and the control (Figure 6b). The concentration of the CAT enzyme in the group treated with 20% biochar increased by 73.80% after 5 days (Figure 6c). The main source of malondialdehyde is oxidative damage. Therefore, the content of MDA in living animals can reflect the degree of oxidative damage in animals. In our study, the content of MDA after 5 days in the 20% treatment group was 398.52% of that in the control group (Figure 6d).

## 4. Discussion

The current findings suggest that biochar influences the behavior of red fire ants and has negative impacts on them. Additionally, biochar induces alterations in the activity of oxidative stress-related enzymes in ants; this effect is dependent on the concentration of biochar. This provides a basis for the prevention and control of red fire ants and the rational application of biochar. This is in common with some studies by others, where there have been some reports showing negative effects of heavy application of biochar. For example, heavy application of biochar reduces the number of mycorrhizal fungal communities in the soil [19], decreases crop germination [20], inhibits plant root growth [21], and reduces plant biomass [22,23], causing changes in individual structure and oxidative stress in earthworms [11], repellence and toxic effects in termites [24], etc. Some studies have shown that biochar can also affect environmental conditions (soil, etc.), resulting in changes in the physical, chemical, or biological properties of the environment, which can indirectly have deleterious effects on organisms [25,26,27]. Also, some volatile toxic substances can be released from biochar and directly affect organisms [28]. Nevertheless, in our current investigation, we employed three replicates with a limited number of treatments. In order to potentially discover variations, it is necessary to augment the number of treatments in the experiment by using moderate quantities of biochar. Consequently, we intend to enhance both the number of treatments and the number of replications in future investigations.

RIFAs, or invasive fire ants, are eusocial insects that reside in expansive colonies. Each nest harbors tens of thousands of worker ants. Individual behavior plays a crucial role in maintaining colony hygiene within the confined nests. The red fire ants pose a formidable challenge due to their ability to swiftly coordinate attacks as a colony. Moreover, the actions of individual ants within the colony can greatly influence the overall survival of the population. Behaviors such as walking, attacking, grasping, and aggregating are the main active abilities embodied in red fire ants; these behaviors affect their feeding and thus their survival. Our present results show that biochar can reduce the walking, attacking, grasping, and aggregating behaviors of red fire ants and the weight of the biochar-treated group also decreased, which may be attributed to the influence of biochar on the feeding of red fire ants. In addition, we also evaluated the content of related oxidative stress enzymes and malondialdehyde in red fire ants after biochar treatment; the results showed that biochar treatment increased the content of SOD, CAT, and POD related enzymes and malondialdehyde. The occurrence of oxidative stress in cells and the development of a series of cascading reactions such as cellular damage are often caused by the difficulty of antioxidant enzymes in scavenging excessive free radicals and the scavenging of ROS is mainly dependent on antioxidant enzymes, the most important of which are superoxide dismutase (SOD) and catalase (CAT), which is suggestive of a large amount of biochar induced oxidative stress in the red fire ants. Biochar is a solid product of high-temperature cracking of biomass and waste pyrolysis [29]; organisms can be directly affected by the discharge of volatile hazardous chemicals from biochar [30]. However, a small portion of pollutants is tightly linked to biochar, making it impossible for plants, microbes, or animals to absorb. These contaminants, when present in an organism, might cause harmful consequences. In addition, biochar can affect the environmental conditions in which it is located, leading to changes in the physical, chemical, or biological properties of the soil environment, which can indirectly have deleterious effects on organisms [8,31]. Chen et al. have demonstrated that excessive biochar application can have a toxic effect on termites by affecting the soil environment [24]. Hale et al. examined the impact of applying biochar derived from maize stover at a temperature of 600 °C to soil that was not contaminated, they investigated how this application affected the *Aporectodea caliginosa* earthworm, using several rates of application ranging from 0.5% to 5.0%. The study revealed that rates between 2% and 5% were responsible for inducing weight loss in *A. caliginosa* [32]. In our experiments, biochar can affect the behavior and physiology of red fire ants through these two pathways. This study contributes to our awareness of the possible toxic effects of biochar-amended soil on organisms, which can be harmed by its unintentional use.

At present, the main measure for the prevention and control of red fire ants is still a chemical control, among which chemical bait baiting is widely used due to its ease of use and more thorough control effect [33]. Red fire ant baits typically consist of pesticide, plant oil lure (such as soybean oil), and an insecticide/lure carrier (commonly corn flour). The production method is rather straightforward. When compared to other pesticide formulations, bait offers the benefits of easy application; a small amount of active chemicals are needed and this effectively addresses the issue of a slow biological control. However, baits also face some drawbacks and shortcomings in their use. For example, under humid conditions, the absorption of moisture by the carrier significantly reduces the effectiveness of the bait, which means that the effectiveness of bait application in areas with high red fire ant prevalence, such as golf courses, parks and lawns, irrigated farmland, and flower nurseries, will be limited [34,35,36]. In addition, the loss of active ingredients due to rainfall will not only reduce the efficacy of the drug but also have an adverse effect on the ecological environment and non-target organisms [37], especially in some water protection zones, densely populated residential areas, and tourist areas, which may pose a threat to human health [38]. In addition, the South China district has the climatic characteristics of perennial high temperature, rain, and high humidity and the precipitation is mainly concentrated in April–September, which is highly coincident with the active period of red fire ants; the prevention and control of red fire ants during the rainy season also pose a great challenge. Therefore, it is of great significance to develop red fire ant prevention and control baits that are suitable for humid conditions and resistant to rainwater washouts for the prevention and control of red fire ants. Nong et al. effectively regulated the rate at which red fire ant baits are released by manipulating the size and shape of the particles using polyvinyl alcohol particles as carriers [39]. Kafle et al. created water-resistant red fire ant baits by encapsulating corn carriers with a pre-existing coating material [40]. Additionally, their team utilized a dry wine trough as a carrier to produce waterproof baits. Chen obtained a patent for a new waterproof coating material made from corn alkyd proteins, which can be used as a carrier for bait. However, there are currently no producers utilizing this innovative coating material [41]. Due to its unique chemical, physical, and biological properties, biochar has become a functional material with multiple uses and is widely used in soil remediation, soil improvement, soil carbon sequestration, and fertilizer carriers [7]. Currently, research on biochar as a pesticide slow-release carrier is expanding and biochar can improve pesticide adsorption capacity on soil, limit pesticide active component loss, and therefore meet the goal of pesticide contamination management [42]. Herrera et al. showed that the incorporation of biochar and bentonite into mirex, imidacloprid, and isoproturon pesticide formulations with sodium alginate as the controlled release material resulted in higher encapsulation rates than formulations containing only sodium alginate as the controlled release material and that larger amounts of biochar resulted in higher encapsulation rates and lower release rates than formulations containing only sodium alginate [43]. Céspedes et al. found that the incorporation of bentonite clay, anthracite, and biochar into alginate-based formulations of phytosaniline and oxacillin effectively reduced the release rates of phytosaniline and oxacillin, respectively, and that biochar was the most effective in slowing down the rate of release, as compared to alginate-based formulations and pesticide-only products with no added adsorbent [44]. Our work demonstrates that biochar has a significant impact on the behavior and physiology of red fire ants, ultimately resulting in their demise. Incorporating prior investigations on biochar’s use as a slow-release pesticide, we can explore the possibility of incorporating biochar into baits in a forthcoming study. This could extend the duration of bait effectiveness and allow for its application in moist and aqueous environments.

## 5. Conclusions

The present study revealed that biochar exhibits a concentration-dependent toxicity and repellant effect on RIFAs. Consequently, we suggest that biochar could be explored as a potential method for controlling red fire ants and preparing bait. RIFAs are not significantly affected by lower concentrations of biochar (*w*/*w*, <5%). Increasing the amounts of biochar will result in the displacement of RIFAs (*w*/*w*, >10%), leading to a decline in their overall well-being, including their weight, behavior, physiology, and survival. Significant quantities of biochar can influence the behavior of red fire ants and trigger alterations in the activity of enzymes associated with oxidative stress. Our study proposes the integration of biochar insecticide with RIFA control as a novel approach for integrated pest management. This combined control technique offers significant ecological, environmental, and economic advantages. Future research will prioritize investigating the processes via which biochar affects the behavior of red fire ants and its potential use in baits.

## Data Availability

All data generated or analyzed during this study are included in this published article.
